# IgE induces proliferation in human airway smooth muscle cells: role of MAPK and STAT3 pathways

**DOI:** 10.1186/1710-1492-9-41

**Published:** 2013-10-17

**Authors:** Naresh Singh Redhu, Lianyu Shan, Duaa Al-Subait, Heather L Ashdown, Hesam Movassagh, Bouchaib Lamkhioued, Abdelilah S Gounni

**Affiliations:** 1 Department of Immunology, Faculty of Medicine, University of Manitoba, 419 Apotex Centre- 750 McDermot Ave, Winnipeg, MB R3E 0T5, Canada; 2 Current address: Department of GI/Nutrition, Boston Children’s Hospital, Harvard Medical School, Boston, MA 02115, USA; 3 UFR de Pharmacie, Universite de Reims, Champenne-Ardene, Reims, France

**Keywords:** Human, Airway remodeling, EdU incorporation, Airway smooth muscle, Syk, MAPK, STAT3

## Abstract

Airway remodeling is not specifically targeted by current asthma medications, partly owing to the lack of understanding of remodeling mechanisms, altogether posing great challenges in asthma treatment. Increased airway smooth muscle (ASM) mass due to hyperplasia/hypertrophy contributes significantly to overall airway remodeling and correlates with decline in lung function. Recent evidence suggests that IgE sensitization can enhance the survival and mediator release in inflammatory cells. Human ASM (HASM) cells express both low affinity (FcεRII/CD23) and high affinity IgE Fc receptors (FcεRI), and IgE can modulate the contractile and synthetic function of HASM cells. IgE was recently shown to induce HASM cell proliferation but the detailed mechanisms remain unknown. We report here that IgE sensitization induces HASM cell proliferation, as measured by ^3^H-thymidine, EdU incorporation, and manual cell counting. As an upstream signature component of FcεRI signaling, inhibition of spleen tyrosine kinase (Syk) abrogated the IgE-induced HASM proliferation. Further analysis of IgE-induced signaling depicted an IgE-mediated activation of Erk 1/2, p38, JNK MAPK, and Akt kinases. Lastly, lentiviral-shRNA-mediated STAT3 silencing completely abolished the IgE-mediated HASM cell proliferation. Collectively, our data provide mechanisms of a novel function of IgE which may contribute, at least in part, to airway remodeling observed in allergic asthma by directly inducing HASM cell proliferation.

## Introduction

Nearly 80% of children and more than 50% of adult asthma is thought to be allergic/immunoglobulin E (IgE)-dependent [1]. Classical dogma defines the allergic reaction in two steps; first when antigen-specific IgE binds to its high affinity Fc receptor (FcεRI) on mast cells and basophils (sensitization step). Next, antigen/allergen binding to specific IgE 'cross-links’ the FcεRI which culminates in various cell activation events such as degranulation, de novo synthesis and secretion of inflammatory mediators, and promotion of cell survival and migration [2]. However, recent studies have established a new paradigm in which IgE sensitization alone can induce a spectrum of effects such as the release of proinflammatory cytokines and chemokines, inhibition of apoptosis or induction of pro-survival effects through activation of various signaling pathways. So far, monomeric IgE has been shown to enhance the survival of mast cells, monocytes, and asthmatic neutrophils [2,3].

Airway smooth muscle (ASM) cells are structural entities of airways which are believed to confer an abnormally exaggerated bronchoconstriction in asthma, the phenomenon commonly known as airway hyperresponsiveness (AHR). Clinically, majority of asthma patients show a significant increase in ASM bundles, likely due to increase in cell number, collectively contributing to airway remodeling [4]. Tissue remodeling due to increased ASM mass in allergic asthma is also known to correlate with AHR in some patients [5]. Although precise mechanisms remain yet to be established, an increase in cell number (hyperplasia) is suggested to be one of the primary factors underlying this increase in ASM mass [4,6]. Molecular studies suggest that mitogen activated protein kinases (MAPK) family and signal transducer and activator of transcription (STAT) 3, besides other pathways, play pivotal role in regulating ASM cell proliferation under various contexts [7,8].

Serum IgE levels have been shown earlier to modulate smooth muscle function. Bronchial hyperresponsiveness was shown to be associated with serum IgE levels [9]. IgE was also shown to cause smooth muscle contractile function through binding to the smooth muscle membrane and subsequent hyperpolarization [10]. We and others have demonstrated previously that human ASM (HASM) cells express a functional tetrameric high affinity FcεRI (αβγ2). IgE/anti-IgE stimulation of HASM induced the release of Th2 and proinflammatory mediators IL-4, -5, -13, TNF, IL-6, CCL11/eotaxin-1, and thymic stromal lymphopoietin (TSLP), and enhanced intracellular calcium ([Ca^2+^]i) mobilization [11,12]. Cumulative evidence has established a critical role of IgE/FcεR interaction in modulation of HASM function and phenotype [12-15]. Although IgE-induced ASM proliferation was reported recently [16], the molecular mechanisms remain unknown. We show here that IgE induces proliferation of ASM cells via MAPK, Akt, and STAT3 signaling pathways; suggesting that IgE may indeed contribute, at least partly, to the development of airway remodeling in allergic asthma.

## Materials and methods

### Reagents

Recombinant human IgE was obtained from Diatec (BioPorto Diagnostics A/S, Denmark). Fetal bovine serum (FBS), sodium pyruvate, trypsin were purchased from HyClone (Logan, UT, USA). 100× L-glutamine, DMEM, Ham’s F-12, trypsin-EDTA, and antibiotics (penicillin, streptomycin) were purchased from Invitrogen Canada Inc. (Burlington, ON, Canada). Platelet-derived growth factor-BB (PDGF-BB) was from R&D Systems, Minneapolis, MN, USA). Rabbit anti-human p38 MAPK mAb, affinity-purified mouse anti-phospho-ERK1/2 (T202/Y204), rabbit anti-human ERK1/2 mAb, affinity-purified rabbit anti-phospho-p38 MAPK (T180/Y182), rabbit anti-total and phospho-specific SAPK/JNK (T183/Y185) Abs, rabbit mAb phospho-Akt (Ser 473) and total-Akt antibody were purchased from Cell Signaling Technology, Inc (Danvers, MA). Mouse mAb anti-phospho-tyrosine STAT3 (Y705) was from BD Biosciences (Mississauga, ON). Affinity purified rabbit anti-total STAT3 antibody and rabbit polyclonal anti-Syk (C-20) antibody were from Santa Cruz Biotechnology, Inc. (Santa Cruz, CA). The p38 MAPK inhibitor, SB-203580; JNK inhibitor, SP-600125; p42/p44 ERK inhibitor, U-0126; and cell-permeable Akt inhibitor VII, TAT-*Akt-in* were purchased from CALBIOCHEM® (EMD Millipore), San Diego, CA, USA. Unless stated otherwise, all other reagents were obtained from Sigma-Aldrich Canada Ltd. (Oakville, ON, Canada).

### Culture and stimulation of HASM cells

HASM cells were prepared and maintained as we have reported earlier [11,17,18]. Written informed consent was obtained from the tissue donors, and this study was approved by the research ethics committee of the University of Manitoba, Winnipeg, Canada. In all experiments, sub-confluent HASM cells were growth arrested and synchronized by serum deprivation for 48 h in Ham’s F-12 medium containing 1× ITS (5 μg/ml human recombinant insulin, 5 μg/ml human transferrin, 5 ng/ml selenium), and antibiotics (100 U/ml penicillin and 100 μg/ml streptomycin). Cells were then stimulated in fresh FBS-free medium with agonists for indicated time periods.

### Manual cell counting and ^3^H-thymidine incorporation to measure HASM cell proliferation

HASM cell proliferation was measured by manual cell counting. Tritiated (^3^H)-thymidine incorporation assay was performed to measure DNA synthesis as a surrogate marker of cell proliferation by following the method of Goncharova and colleagues [19] with minor modifications. Briefly, ASM cells were seeded in 24-well tissue culture plates (3 × 10^4^ cells/well) to grow to about 70% confluency in a 37°C humidified 5% CO_2_ incubator. Cells were serum-deprived in Ham’s F12 containing 1× ITS media for 48 h to growth-arrest and synchronize them. Fresh F12 containing 1× ITS was added and cells were stimulated with graded doses of IgE and other mitogens for 16 h. 10% FBS or PDGF-BB (10 ng/ml) was used (following dose titration) as a positive control. After 16 h, *methyl*-^3^H-thymidine (Specific activity 20 Ci [740 GBq]/mMole, >97%; Perkin Elmer, Woodbridge, ON, Canada) was added at a final concentration of 2 μCi/ml and cells were incubated at 37°C for 24 h. Subsequently, ASM cells were rinsed in PBS three times before adding 0.1 ml 0.05% trypsin-EDTA for 15 minutes at 37°C for lysis, followed by addition of 0.1 ml ice-cold 20% trichloroacetic acid (TCA) for 20 minutes at 4°C to precipitate the DNA. Precipitated DNA was then carefully transferred to 96-well plates to facilitate its absorption on 96-well format glass fibre filter mats (Wallac, Turku, Finland) using Tomtec Harvestor 96 (Tomtec Inc., Hamden, CT, USA). Filter mats were air-dried and counted in liquid scintillation counter. In some experiments, MAPK inhibitors were used for one hour prior to IgE stimulation. Experiments were performed in triplicate and the data was presented as mean ± SEM of counts per minute (cpm).

### EdU-incorporation assay for HASM cell proliferation

HASM cell proliferation was additionally measured by using Click-it EdU Proliferation kit (Invitrogen) by following the manufacturer’s instructions. Briefly, sub-confluent 48 h serum-starved ASM cells were stimulated with graded doses of IgE (5-25 μg/ml) and PDGF (10 ng/ml) for 16 h following which cells were allowed to incorporate EdU (5-ethynyl-2′-deoxyuridine; 5 μM) for 24 h and then trypsinized and fixed. Fixed cells were immediately processed for staining with Click-it EdU detection reagent conjugated with Alexa Fluor 488, and cell nuclei were stained with DAPI. EdU-positive cells were visualized by using flow cytometry and are presented as % proliferating population on right side of the histogram.

### Western blotting to assess MAPK and STAT3 phosphorylation

IgE-induced ASM signaling pathways were studied by performing Western blotting for phosphorylated MAPK and STAT3, as described earlier [20]. Intensity of phosphorylation was assessed by performing densitometry analysis using AlphaEaseFC (FluorChem 8800) Software. The data was presented as fold increase in the ratio of phospho and total compared to time zero.

### Lentivirus-mediated STAT3-shRNA transduction in HASM cells

Lentiviral transduction of Syk-short hairpin (sh) RNA (clone ID: V2LHS_153702) and STAT3-short hairpin (sh) RNA (clone ID: V2LHS_262105) in HASM cells was performed as described earlier [21]. Mock and lentiviral-Syk- or lentiviral-STAT3-shRNA-transduced HASM cells were cultured in presence of IgE (10 μg/ml), PDGF-BB (10 ng/ml), FBS (10%), or medium alone; and cell proliferation was assessed by ^3^H-thymidine incorporation assay.

### Statistical analysis

Statistical analysis was performed by using GraphPad Prism Software Version 3.02 for Windows (GraphPad Software, San Diego, CA, USA). Data between groups was compared by using student’s unpaired *t* test. P values <0.05 were considered statistically significant.

## Results

### IgE induces DNA synthesis and proliferation in HASM cells

**Figure 1:**
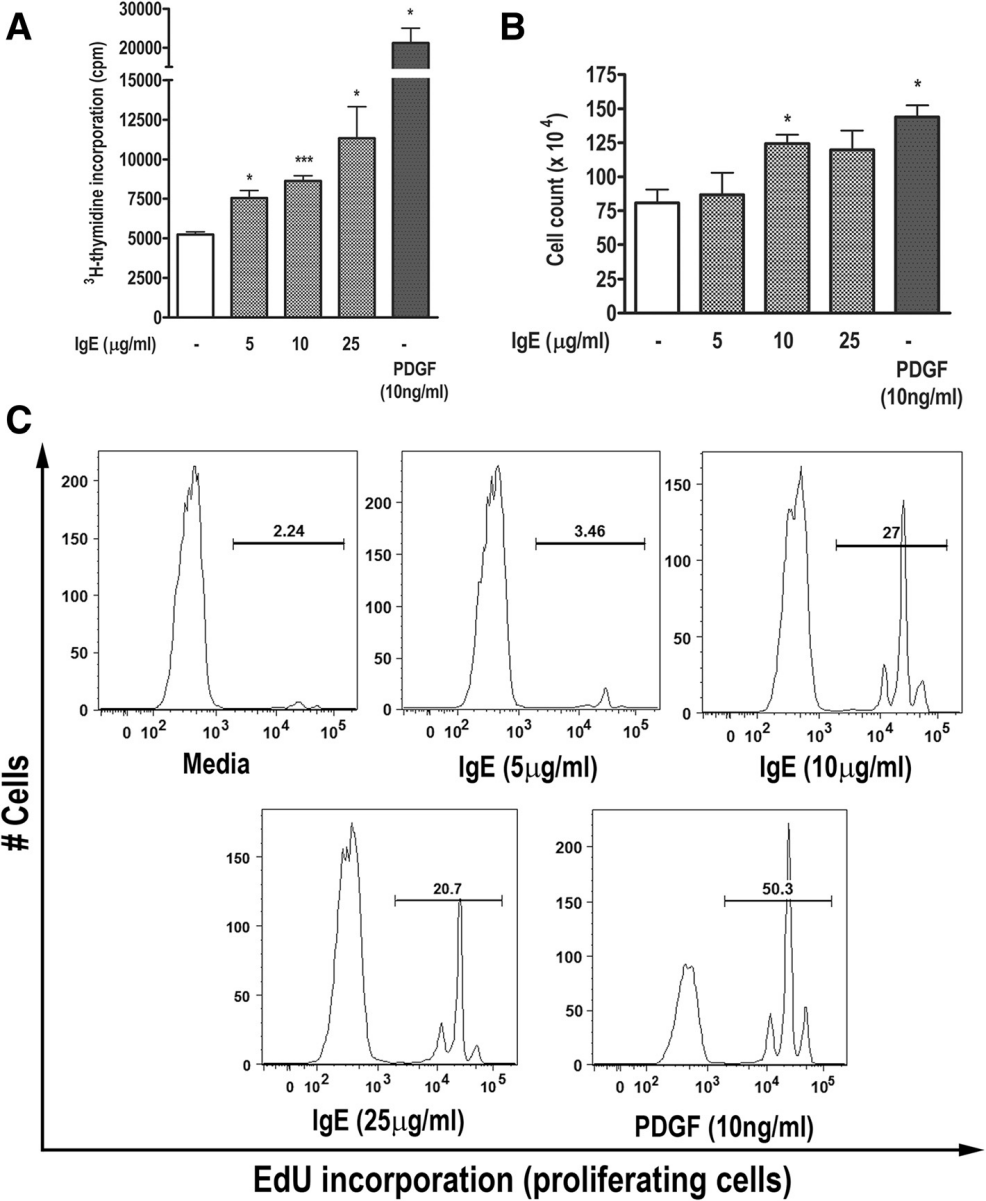
**IgE induces DNA synthesis and cell proliferation in HASM cells. (A)** Serum-starved HASM cells were stimulated with IgE (5–25 μg/ml), PDGF-BB (10 ng/ml), or left unstimulated. ^3^H-thymidine incorporation was measured by liquid scintillation counting and presented as cpm. **(B)** IgE or PDGF-BB-stimulated cells were trypsinized after 48 h and counted by hemocytometer. **(C)** HASM cells stimulated similar to **(A)** were subjected to EdU-incorporation assay. EdU positive cell populations from a representative experiment are shown. n = 3; *p < 0.05, ***p < 0.001 compared to unstimulated control.

To test the mitogenic potential of IgE on human ASM cells, we performed ^3^H-thymidine incorporation assay. While IgE did not affect cell survival (data not shown); as shown in Figure 1A, IgE induced *de novo* DNA synthesis in HASM cells (n = 5, p < 0.05). As expected, PDGF induced prominent increase in DNA synthesis and served as positive control [8,18]. We further validated the IgE-induced ^3^H-thymidine incorporation data by using hemocytometer-based cell counting. IgE-induced thymidine incorporation appeared to have translated into increase in cell number compared to control (Figure 1B, n = 4, p < 0.05), suggesting that IgE is able to induce DNA synthesis and subsequent proliferation in HASM cells (Figure 1A, B).

In addition, we confirmed the proliferative effect of IgE on HASM cells by using EdU incorporation. As shown in Figure 1C, IgE clearly induced HASM cell proliferation, in almost similar manner to ^3^H-thymidine incorporation and manual cell counting (Figure 1). Therefore, our data suggest that IgE can induce HASM cell proliferation.

### Lentivirus-mediated Syk inhibition abrogates IgE-induced HASM proliferation

**Figure 2:**
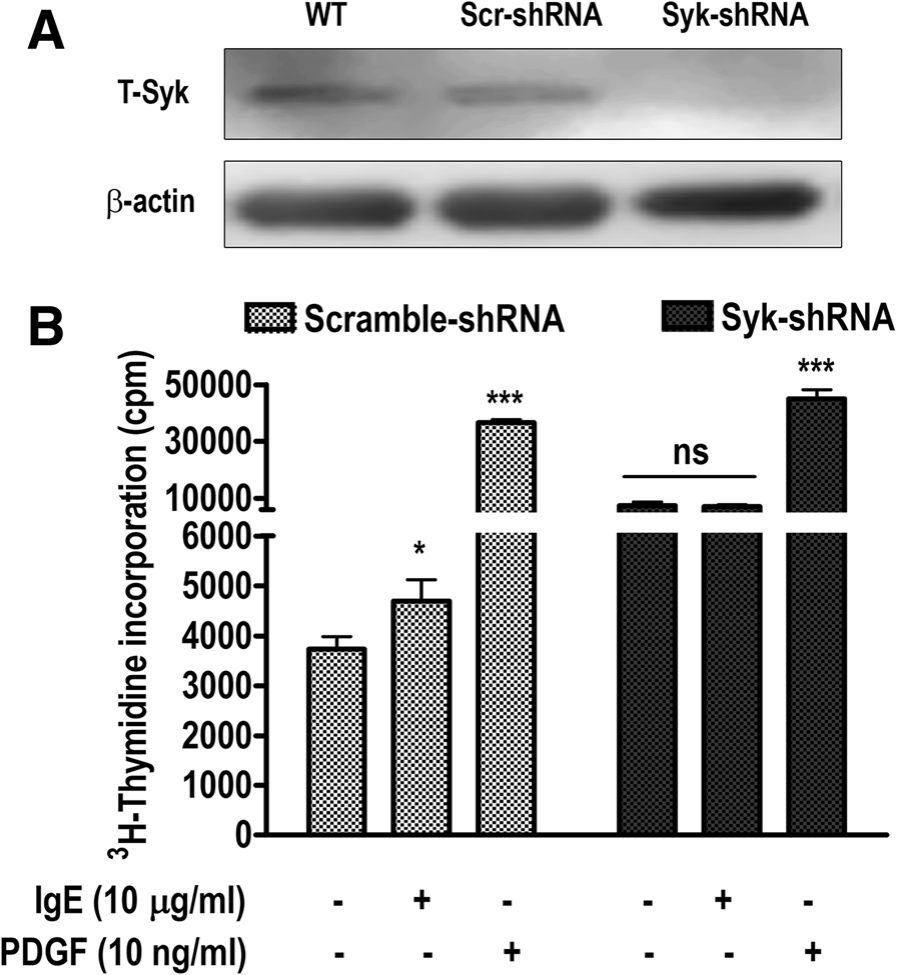
**IgE-induced HASM cell proliferation requires Syk activity. (A)** HASM cells were stably transduced with pseudotyped lentiviral vectors expressing Syk-shRNA or non-specific scramble-shRNA. Syk expression was assessed by Western blotting as described in *Material and Methods* section. **(B)** Syk- or scramble-shRNA-transduced HASM cells were stimulated with IgE (10 μg/ml), PDGF-BB (10 ng/ml), or left unstimulated. 3H-thymidine incorporation was measured as a marker of cell proliferation as in Figure 1A. n = 3, *p < 0.05, ***p < 0.001, and ns, non-significant compared to unstimulated control.

FcεRI activation leads to a spectrum of signaling events in inflammatory cells, starting with phosphorylation of Lyn kinase followed by recruitment and phosphorylation of Syk. Activation of Syk then serves as an indispensable mechanism of downstream propagation of signals leading to the activation of various kinases, transcription factors, mediator release, and survival [2,22,23]. This suggests that inhibition/silencing of Syk might be a useful strategy to validate the role of Syk and FcεRI pathway in IgE-induced HASM cell proliferation. To test this, we utilized the lentiviral-mediated Syk inhibition strategy, which we have reported earlier in IgE-induced mediator release in HASM cells [15,17]. HASM cells were stably transduced with pseudotyped lentiviral vector expressing specific Syk-shRNA. Mock and scramble sequence were used as negative controls. As reported earlier [15,17], more than 95% of HASM cells were transduced by turbo-GFP signal positivity by FACS analysis (data not shown). Lentiviral-Syk-shRNA but not control scramble-shRNA transduction resulted in a highly significant and reproducible decrease in Syk expression, as shown by Western blotting (Figure 2A). We then used these lentiviral-transduced cells and stimulated them with IgE and PDGF. As shown in Figure 2B, scramble-shRNA-transduced HASM cells demonstrated a significant increase in thymidine incorporation (p < 0.05, n = 3) similar to the wild-type cells (Figure 1A). However, Syk-shRNA-transduced cells lost the effect of IgE (p > 0.05, non-significant). PDGF consistently showed highly significant (p < 0.001) thymidine incorporation in both scramble and Syk-inhibited HASM cells (Figure 2B). These results suggest that IgE-induced proliferation requires the function of Syk, a key signaling pathway in FcεRI activation.

### IgE activates multiple signaling pathways in HASM cells

**Figure 3:**
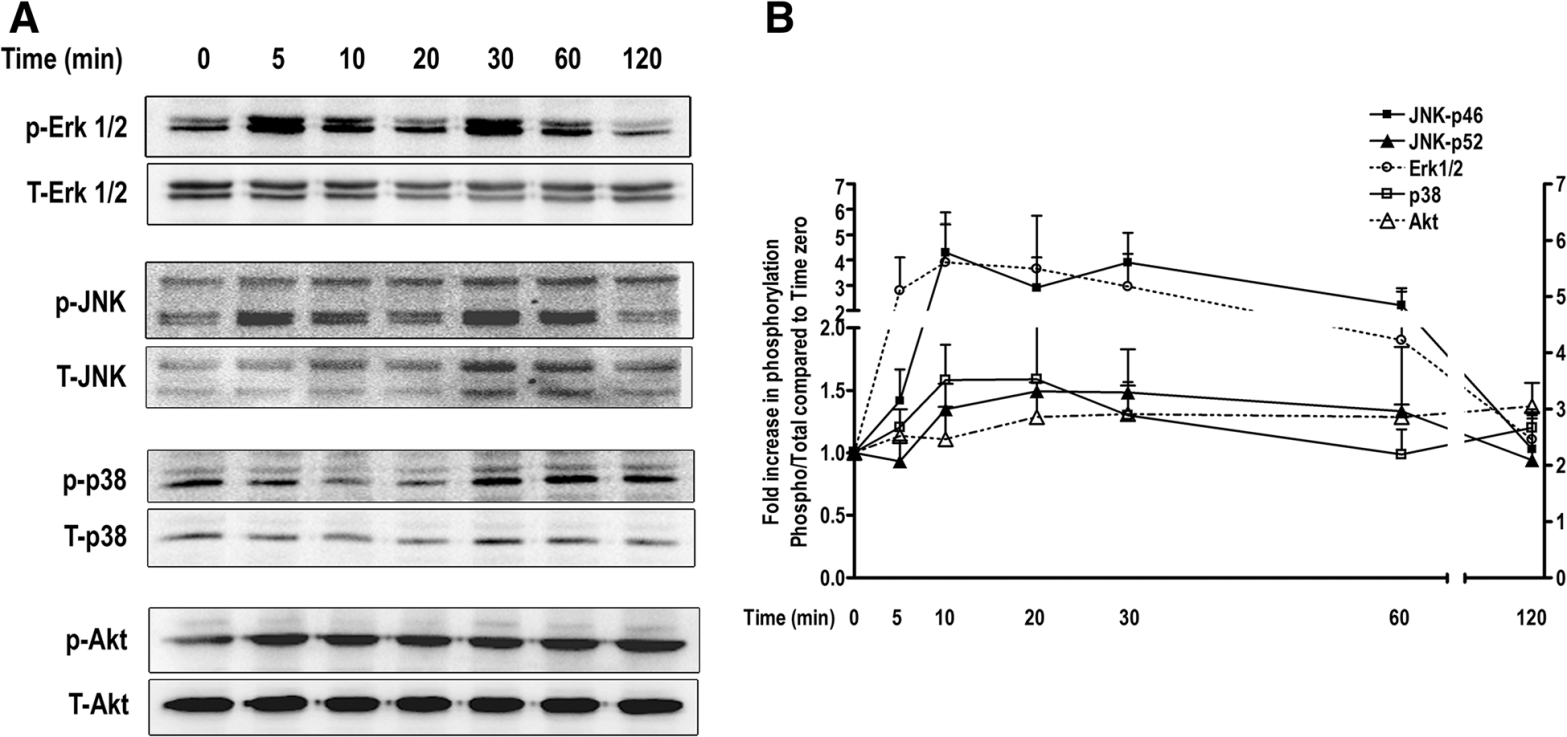
**IgE induces MAPK and Akt phosphorylation in HASM cells. (A)** Phosphorylation of MAPKs (ERK1/2, p38, and JNK) and Akt were assessed by immunoblotting from total cell lysates of IgE-stimulated HASM cells as described in *Materials and Methods* section. **(B)** Phosphorylation intensity was measured be densitometry analysis and expressed as fold increase over time zero of the ratio of phospho over total levels (n = 3).

To understand the downstream molecular signaling pathways involved in IgE-induced HASM cell proliferation, we assessed the phosphorylation of MAPK and Akt by performing Western blot analysis on HASM cell lysates stimulated with IgE for 0–120 min. Western blotting revealed a significant JNK phosphorylation at 20–30 min, Erk1/2 at 60 min, p38 at 120 min, and Akt at 60 min (n = 3, Figure 3A, B). In summary, IgE phosphorylates MAPK and Akt kinases in HASM cells which may play a role in IgE-induced cell proliferation.

### MAPK inhibitors abrogate the IgE-induced HASM cell proliferation

**Figure 4:**
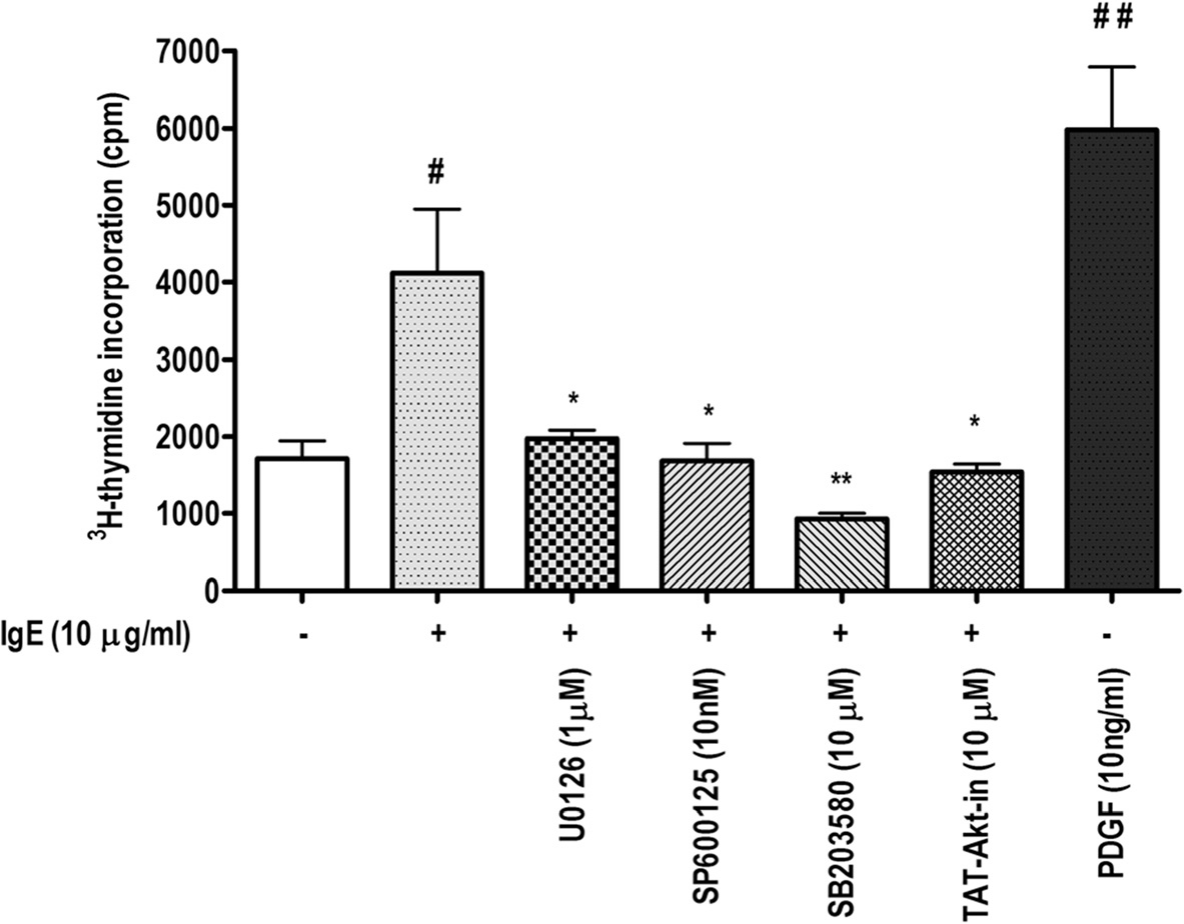
**MAPK and Akt inhibitors abrogate the IgE-induced HASM cell proliferation. **^3^H-thymidine incorporation assay was performed in serum-starved HASM cells stimulated with IgE for 24 h following pretreatment for 1 h with inhibitors of ERK 1/2 (1 μM, U0126), JNK (10 nM, SP600125), and p38 MAPK (10 μM, SB203580), and Akt (10 μM, TAT-*Akt-in*). #p < 0.05, ##p < 0.01 compared with unstimulated control. *p < 0.05, **p < 0.01, ns, non-significant compared with IgE-stimulated cells.

We then confirmed the involvement of different MAPKs in IgE-induced HASM cell proliferation by using specific MAPK inhibitors. The dose of various inhibitors was first optimized to find the dose that inhibits IgE-induced cell proliferation without inducing a noticeable cytotoxicity (data not shown). Figure 4 shows that IgE-induced HASM cell proliferation was inhibited significantly (p < 0.05, n = 3) upon pre-incubation for one hour with inhibitors of Erk1/2 (1 μM, U0126), JNK (10 nM, SP600125), p38 (10 μM, SB203580), and Akt (10 μM, TAT-*Akt-in*). DMSO vehicle control did not show any effect on HASM cell proliferation (data not shown). In conclusion, IgE-induced HASM cell proliferation involves the activation of Erk1/2, p38, JNK MAPK, and Akt kinases.

### STAT3 is critical in IgE-induced HASM cell proliferation

**Figure 5:**
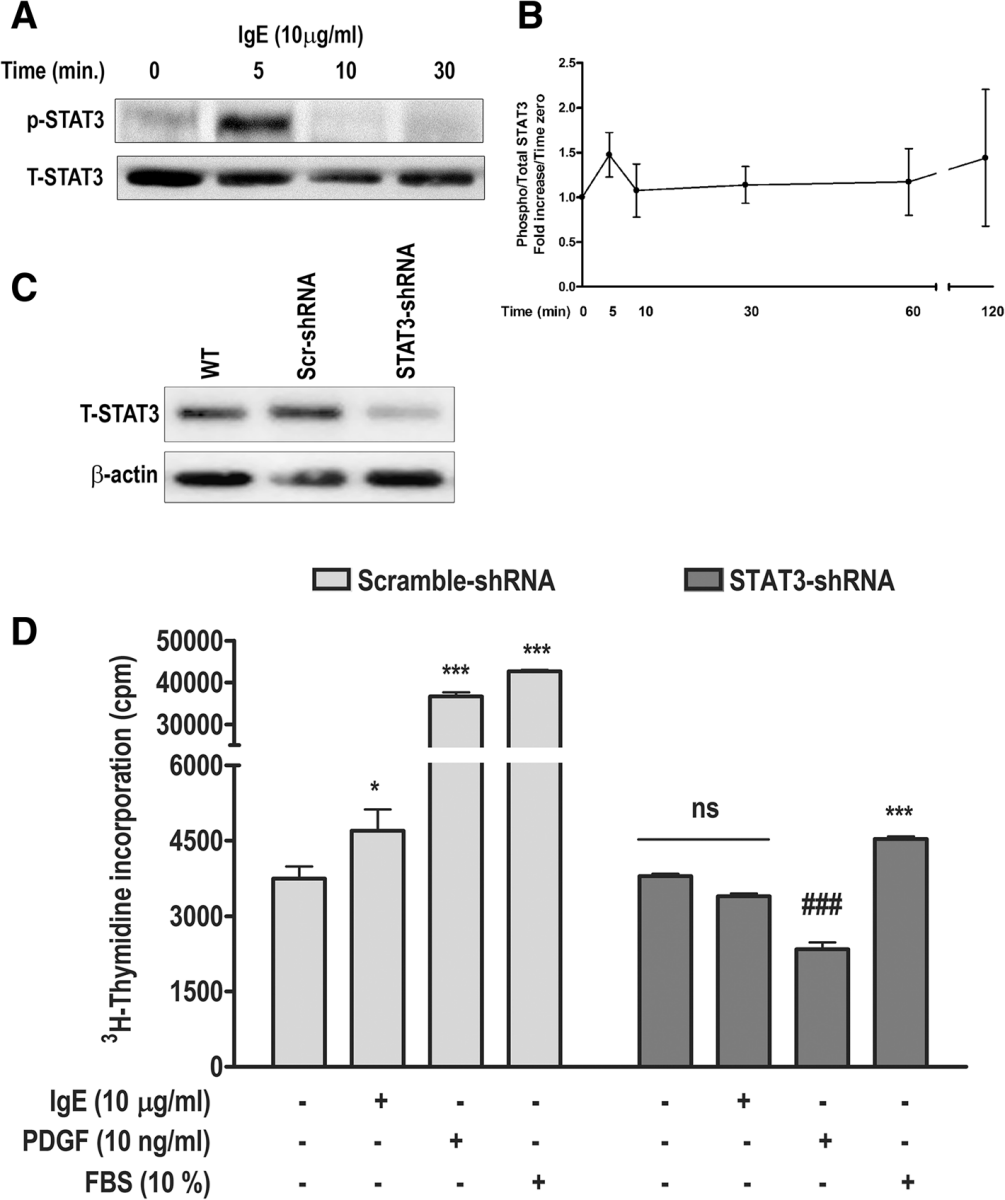
**Lentivirus-mediated STAT3-inhibition abrogates IgE- and PDGF-induced proliferation in HASM cells. (A)** IgE-induced STAT3 phosphorylation was determined by Western blotting. **(B)** Summary of four experiments to assess IgE-induced STAT3 phosphorylation. **(C)** Expression of total STAT3 in wild-type (WT or non-transduced), scramble-shRNA, and STAT3-shRNA–transduced HASM cells was analyzed by Western blotting. **(D)** Stably lentivirus-transduced HASM cells were stimulated with IgE, PDGF-BB, or 10% FBS, and ^3^H-thymidine incorporation was performed. Data represents mean ± SEM; *p < 0.05, ***p < 0.001, ns, non-significant compared with respective unstimulated cells. ###p < 0.001 compared with STAT3-shRNA-transduced unstimulated cells.

STAT3 activation is indispensable in HASM cell proliferation in response to PDGF [8]. Interestingly, monomeric IgE induces STAT3 phosphorylation in murine bone marrow-derived mast cells and rat basophilic leukemia cells, and induce the transcription of genes important in cell survival [24]. With these reports in consideration, we first sought to determine whether IgE is able to phosphorylate STAT3 in HASM cells. A representative blot in Figure 5A and summary of 4 experiments in Figure 5B show that IgE indeed induced STAT3 phosphorylation in HASM cells. To confirm its role in HASM cell proliferation, we employed lentiviral vector-mediated STAT3 silencing approach [20]. HASM cells were stably transduced with pseudotyped lentiviral vector encoding specific STAT3-shRNA. Mock and scramble sequence served as controls. More than 95% of HASM cells were transduced as observed by turbo-GFP signal by FACS analysis (data not shown). Lentiviral-STAT3-shRNA transduction resulted in a noticeable decrease in STAT3 expression compared to WT or scramble-shRNA transduction controls (Figure 5C) [21]. Both scramble-shRNA- and STAT3-shRNA-transduced HASM cells were stimulated with IgE and PDGF to analyze thymidine incorporation. Since PDGF-induced mitogenic signaling requires STAT3 expression [8], 10% FBS was used as an additional positive control in this experiment. As expected, scramble-shRNA-transduced HASM cells showed a normal and statistically significant response to IgE (p < 0.05), PDGF, and 10% FBS (p < 0.001) compared with unstimulated control (Figure 5D). However, the effect of IgE was completely abrogated in STAT3-shRNA transduced cells, and so was the effect of PDGF, also confirming the previous reports [8]. On the other hand, although 10% FBS showed increased thymidine incorporation in STAT3-shRNA-transduced cells, the effect was much less pronounced when compared with scramble-shRNA-transduced HASM cells (Figure 5D). This is consistent with the observation by other groups [8], and suggests that the serum components may also require STAT3 activation to induce mitogenic signaling in HASM cells. In summary, our data suggest that IgE-induced STAT3 activation plays a critical role in HASM cell proliferation.

## Discussion

We report in this study that IgE sensitization induces DNA synthesis and proliferation in HASM cells through the activation of Syk, and signaling Erk 1/2, p38, JNK MAPK, and Akt kinases. Lentivirus-shRNA-mediated experiments showed that STAT3 activation is indispensable for IgE-induced HASM cell proliferation. Collectively, we show for the first time that IgE sensitization can directly induce human ASM cell proliferation which may contribute, at least partly, to the airway remodeling in allergic asthma.

Serum IgE levels were shown to affect ASM cell function and tend to correlate with AHR [9]. Cumulative data in last decade has defined a direct role of IgE in ASM cell activation. We and others have shown that FcεRI activation by IgE/anti-IgE incubation leads to enhanced release of pro-asthmatic cytokines (IL-4, -5, -13, TNF, IL-6, and TSLP), eosinophil-attracting CCL11/eotaxin-1 chemokine; and a rapid and transient increase in ([Ca^2+^]i) mobilization, altogether suggesting a critical role of this pathway in airway inflammation and hyperresponsiveness [11,12,14,15]. Importantly, blocking of FcεRI led to abrogation of IgE-induced HASM cell synthetic functions [11]. Moreover, TNF and IL-4 can augment FcεRI expression and amplify IgE-induced release of chemokines including CCL11/eotaxin-1, CCL5/RANTES, CXCL8/IL-8, and CXCL10/IP-10 [17]. Although Xia et al. were unable to demonstrate the expression of FcεRI in ASM cells [25], possible explanations for this discrepancy were discussed recently [26]. Furthermore, other groups have shown that IgE/anti-IgE treatment of HASM cells induce modest levels of matrix metalloprotease 1 (MMP-1) production which may contribute to airway inflammatory and remodeling responses [27]. Finally, a clinically-proven anti-IgE monoclonal antibody Omalizumab abrogated the IgE-induced mediators of asthma relevance such as IL-4, IL-6, IL-8, and TNF [12]. The current study extends the function of IgE on HASM cells by suggesting a direct mitogenic effect which may have critical consequences on airway tissue remodeling. Interestingly, IgE induced significantly higher cell proliferation in ASM cells obtained from asthma compared to that from normal individuals [16]. *In vivo*, anti-IgE treatment decreased the thickness of ASM layer compared with the ovalbumin (OVA)-challenged mice [28], suggesting that IgE could be one of the factors inducing ASM remodeling *in vivo*. Although the low affinity (FcεRII/CD23) receptor has also been described in ASM cells with enhanced signal in ASM tissue from asthma [29], and Roth et al. have suggested the involvement of both FcεRII/CD23 and FcεRI in IgE-induced ASM remodeling [16]; presently observed proliferative effect of IgE appear to primarily involve FcεRI since the lentiviral shRNA-mediated inhibition of spleen tyrosine kinase (Syk), a signature kinase in FcεRI signaling [22], abolished the IgE-induced HASM proliferation. However, the role of FcεRII/CD23 in this process cannot be denied. Of note, Syk inhibition in our study led to increase in basal ASM cell proliferation. Previous studies have shown that Syk regulates proliferation and migration in non hematopoietic cells. In Syk knockout mice, aberrant development of the blood and lymphatic vessels is due to abnormal endothelial cell proliferation and migration [30,31]. Furthermore, Syk also regulates breast epithelial cell proliferation, migration, and differentiation. In fact, the absence of Syk correlated with increased aggressiveness and metastases of the tumors. In humans, ductal cell carcinomas [32] and in vitro studies have shown that reconstitution of Syk expression abrogated the abnormal cell proliferation observed in a cancerous breast epithelial cell line [33]. Therefore, the relatively higher basal cell proliferation in our Syk-silenced HASM cells may be attributed to the fundamental nature of Syk in regulating the cell proliferation.

STAT3 has been shown earlier to regulate allergic response in asthma. Particularly, epithelial STAT3 was identified as a critical regulator of allergen-induced inflammation and AHR in a murine model of asthma [34], IL-17A-induced STAT3 activation led to CCL11/eotaxin-1 production in HASM [21], and PDGF-induced STAT3 mediated the proliferation in HASM cells [8]. Besides PDGF, IgE was shown to induce STAT3-dependent transcription of pro-survival genes in mast cells [24]. We observed a clear phosphorylation of STAT3 in response to IgE, the functional role of which was confirmed by lentivirus-shRNA-mediated STAT3 inhibition that completely abrogated the IgE-induced HASM cell proliferation. Interestingly, although both PDGF and IgE activated STAT3, we did not observe any synergy between both in modulating HASM cell proliferation (data not shown).

Although IgE-induced signaling pathways are well characterized in inflammatory cells, there is limited information on this area in HASM cells. MAPK family is fundamental in regulating multiple cell functions such as cytokine expression, proliferation, and apoptosis. Although Erk1/2 and p38 MAPK were shown to mediate IgE-induced proinflammatory gene expression in HASM recently [12], Akt was observed to be activated in response to IgE for the first time in HASM. However, the role of Akt (also called protein kinase B) is well defined in HASM cell mitogenic signaling [35]. The p38 MAPK is also known for its pro-remodeling function in allergic asthma [36]. Furthermore, studies show that MAPK (at least ERK1/2) can modulate the STAT3 activation in HASM [21,37]. However, it is unclear and deserves further investigation whether MAPK and STAT3 signaling pathways cross-talk to induce IgE-mediated proliferation. Collectively, IgE induced the activation of multiple signaling pathways (ERK1/2, p38, JNK MAPK and STAT3) which suggests a complex network of signaling pathways in mediating IgE-FcεR signaling in HASM cells. Further studies are underway to delineate these cross-regulatory interactions in HASM cell proliferation.

Mechanistically, there is sufficient evidence from past decade to convince that the 'IgE sensitization/monomeric IgE exposure’ of FcεRI on inflammatory cells itself can activate multiple signaling pathways; induce a plethora of proinflammatory mediators release and cell survival factors, and subsequent repression of apoptosis [38]. Interestingly, IgE-induced survival or cytokine release does not necessarily require receptor aggregation and merely receptor occupancy can induce these effects [39-42]. Nonetheless, the role of FcεRI cross-linking in conferring pro-survival effect has been a matter of debate. While two initial reports [39,40] suggested the lack of cross-linking, Xiang et al. [43] argued for FcεRI cross-linking-mediated degranulation in mast cell survival. IgE induced monocyte survival in both instances (aggregation or no aggregation), while mast cells and asthmatic neutrophils showed IgE-mediated survival without FcεRI cross-linking or aggregation [3,41]. These findings are supported by *in vivo* observations where IgE can promote immune sensitization to hapten in the skin, without the need of antigens [44]. Not only monoclonal IgE, a recent report suggest that the polyclonal IgE from human atopic dermatitis patients can induce survival effects and cytokine release in human cord blood-derived mast cells, a finding that is clinically more relevant [45]. Of note, HASM cells have been shown to be activated by both 'sensitization alone’ [12,15,17] and 'cross-linking’ [11,27] models. Whether the currently observed mitogenic effects of IgE on HASM cell require cross-linking/aggregation is not clear. However, the cross-linking of FcεRI-bound IgE with anti-IgE antibodies from various sources did not further augment the HASM cell thymidine incorporation in our study (data not shown). In conclusion, our data suggest that the mitogenic effect of IgE on HASM cells may occur through simple receptor occupancy without cross-linking.

Omalizumab, the clinically approved anti-IgE antibody blocks the interaction of IgE with FcεRI and has shown clinical benefits in controlling allergic inflammation, and offered improvement in asthma symptoms, reduced frequency of asthma exacerbations, and significantly lowered the use of inhaled corticosteroids [46,47]. Finally, the proposal that IgE can induce ASM remodeling is bolstered by two recent clinical studies wherein clinical anti-IgE antibody treatment significantly reduced the airway wall thickness and airway inflammation in severe allergic asthma [16,48,49]. Importantly, anti-IgE therapy may not be the best approach for clinical benefit since IgE already bound to mast cells and basophils and residual IgE can still trigger cell activation. Blocking the FcεRI may be a theoretically better approach. Recent studies have showed that a novel FcεRI-mimetic peptide E (PepE) can block IgE binding to FcεRI and can prevent anaphylaxis in WT mice but has no capacity of blocking anaphylaxis in IgE KO mice that was given IgE before treatment [50]. This suggests that PepE can block the binding of free IgE to FcεRI but cannot compete with the receptor for already bound IgE *in vivo*. In conclusion, blocking the IgE-FcεR interaction, not only on inflammatory cells but also on the airway structural (HASM) cells should be considered as a novel tool to inhibit allergic sensitization-mediated airway remodeling in asthma.

## Competing interests

The authors declare that they have no competing interests.

## Authors’ contributions

NSR and ASG conceived the study, NSR performed the experiments, analyzed the data, and wrote the manuscript; LS, DA, HLA, HM performed the experiments; BL provided critical scientific input; ASG provided the resources, and provided editorial and scientific input on manuscript. All authors read and approved the final manuscript.
